# Dynamics of Serum Tumor Markers Can Serve as a Prognostic Biomarker for Chinese Advanced Non-small Cell Lung Cancer Patients Treated With Immune Checkpoint Inhibitors

**DOI:** 10.3389/fimmu.2020.01173

**Published:** 2020-06-10

**Authors:** Zhibo Zhang, Fang Yuan, Runzhe Chen, Ye Li, Junxun Ma, Xiang Yan, Lijie Wang, Fan Zhang, Haitao Tao, Dong Guo, Zhiyue Huang, Sujie Zhang, Xiaoyan Li, Xiaoyu Zhi, Xiangwei Ge, Yi Hu, Jinliang Wang

**Affiliations:** ^1^Department of Oncology, The First Medical Center of Chinese PLA General Hospital, Beijing, China; ^2^Medical School of Chinese PLA, Beijing, China; ^3^The 78th Group Army Hospital of Chinese PLA, Mudanjiang, China; ^4^Departments of Thoracic/Head and Neck Medical Oncology and Genomic Medicine, The University of Texas MD Anderson Cancer Center, Houston, TX, United States; ^5^Department of Radiotherapy, The First Medical Center of Chinese PLA General Hospital, Beijing, China; ^6^BeiGene (Shanghai) Co., Ltd., Shanghai, China

**Keywords:** non-small cell lung cancer, serum tumor markers, Chinese patients, immune checkpoint inhibitors, prognostic biomarker

## Abstract

**Background:** Serum tumor markers carcinoembryonic antigen (CEA), cancer antigen 125 (CA125), cytokeratin 19 fragment (CYFRA21-1) and squamous-cell carcinoma-related antigen (SCC-Ag) are routinely used for monitoring the response to chemotherapy or targeted therapy in advanced-stage non-small cell lung cancer (NSCLC), however their role in immunotherapy remains unclear. The aim of this study was to investigate whether dynamics of these serum markers were associated with the efficacy and prognosis of Chinese late-stage NSCLC patients treated with programmed cell death-1/programmed cell death ligand-1 (PD-1/PD-L1) inhibitors.

**Methods:** We initiated a longitudinal prospective study on advanced NSCLC patients treated with PD-1/PD-L1 inhibitors in Chinese PLA general hospital (Beijing, China). Blood samples of baseline and after 6 weeks' treatment were collected. CT scan were used by all patients to evaluate treatment efficacy according to RECIST 1.1. Serum tumor markers levels were measured with an electrochemical luminescence for SCC-Ag and with a chemiluminescent microparticle immunoassay for serum CEA, CA125, and CYFRA21-1. At least 20% decreases of the biomarkers from baseline were considered as meaningful improvements after 6 weeks of treatment with immune checkpoint inhibitors (ICIs). Optimization-based method was used to balance baseline covariates between different groups. Associations between serum tumor biomarker improvements and objective response rate (ORR), progression-free survival (PFS), and overall survival (OS) were analyzed.

**Results:** A total of 308 Chinese patients with advanced NSCLC were enrolled in the study. After balancing baseline covariates, patients with meaningful improvements in <2 out of 4 biomarkers (CEA, CA125, CYFRA21-1, and SCC-Ag) was ended up with lower ORR (0.08 vs. 0.35, *p* < 0.001), shorten PFS (median: 5.4 vs. 12.5 months, *p* < 0.001), and OS (median: 11.7 vs. 25.6 months, *p* < 0.001) in the total population. Subgroup analysis of patients with adenocarcinoma revealed that patients with meaningful improvements in <2 out of 4 biomarkers had significant lower ORR (0.06 vs. 0.36, *p* < 0.001), shorten PFS (median: 4.1 vs. 11.9 months, *p* < 0.001), and OS (median: 11.9 vs. 24.2 months, *p* < 0.001). So as in patients with squamous cell carcinoma, meaningful improvements in at least 2 out of 4 biomarkers were linked to better ORR (0.42 vs. 0.08, *p* = 0.014), longer PFS (median: 13.1 vs. 5.6 months, *p* = 0.001), and OS (median: 25.6 vs. 10.9 months, *p* = 0.06).

**Conclusions:** The dynamic change of CEA, CA125, CYFRA21-1, and SCC-Ag from baseline have prognostic value for late-stage NSCLC patients treated with PD-1/PD-L1 inhibitors. Decrease of associated biomarkers serum levels were associated with favorable clinical outcomes.

## Introduction

Lung cancer is the leading cause of cancer-related deaths worldwide (1, 2). As the most common subtype of lung cancer, non-small cell lung cancer (NSCLC) accounts for 80–85% of the total cases. Over 60% of the NSCLC patients present with locally advanced or metastatic diseases at the time of diagnosis, and surgical resection may not be a treatment option (3). For these patients, although chemotherapy or targeted therapy has improved clinical outcomes in certain subtypes of lung cancer, up to 90% of patients inevitably relapse with the 5-year survival rate below 20% (4–6).

The emergence of immune checkpoint blockade (ICB) therapy targeting programmed cell death-1/programmed cell death ligand-1 (PD-1/PD-L1) have revolutionized the treatment of NSCLC, with large number of clinical trials demonstrating their increased effectiveness (7–10). Unfortunately, response rate is only ~20% for advanced NSCLC in unselected populations, thus biomarker development remains critical to avoid ineffective treatments (11). PD-L1 expression and tumor mutation burden (TMB) are the most studied and validated predictors of clinical benefit in NSCLC patients with ICB therapy (12–15), while their roles are still controversial (7, 16–19). Moreover, detecting these biomarkers usually requires and invasive procedures followed by pathological assessment or even complicated and expensive methodologies such as the next generation sequencing (NGS). Therefore, non-invasive method and convenient biomarkers with relatively low cost are urgently needed.

Serum carcinoembryonic antigen (CEA), cancer antigen 125 (CA125), cytokeratin 19 fragment (CYFRA21-1), and squamous-cell carcinoma-related antigen (SCC-Ag) might be relevant for the prognosis of patients and have been widely used as biomarkers predicting the efficacy of chemotherapy or targeted therapy in NSCLC patients (20–27). However, their roles and post-treatment changes from baseline in advanced NSCLC treated by immune checkpoint inhibitors (ICIs) remains unclear. The aim of this study was to investigate whether dynamics of serum tumor markers were associated with the efficacy and prognosis of Chinese late-stage NSCLC patients treated with ICIs.

## Methods

### Study Design

This observational study was performed in a real-life clinical practice setting. A total of 308 consecutive NSCLC patients from stage IIIB to IV receiving PD-1/PD-L1 checkpoint inhibitors were prospectively enrolled in Chinese PLA general hospital (Beijing, China) from January 2015 to January 2019. ICIs were treated for at least 6 weeks, and serum biomarkers (CEA, CA125 CYFRA21-1, and SCC-Ag) were measured at ICIs treatment initiation and after 6 weeks. During treatment, response was evaluated at least once.

The efficacy of immunotherapy was assessed according to Response Evaluation Criteria in Solid Tumors (RECIST) version 1.1 (28), including complete response (CR), partial response (PR), stable disease (SD), and progressive disease (PD). ORR was defined as the percentage of patients who have ever achieved a CR or PR since the first ICIs treatment. The time interval between date of commencement of PD-1/PD-L1 inhibitors treatment and date of disease progression or death (PFS) or death alone (OS) was calculated for each patient. The data cut-off date was Oct 6, 2019.

The baseline covariates including age, gender, histological type, clinical stage, smoking history, Eastern Cooperative Oncology Group Performance Status (ECOG PS), metastatic sites (lung, liver, and brain), radiotherapy, treatment (monotherapy or combination therapy), and prior lines of therapy (one line, two lines, and at least three lines) were collected. Lab test results including hemoglobin, white blood count, neutrophil, lymphocyte, monocyte, lactate dehydrogenase, platelet, and albumin were also routinely recorded.

### Specimen Collection and Tumor Markers Assay

Blood samples were collected before the first ICIs treatment and after 6 weeks. Serum levels of CEA, CA125, and CYFRA21-1 were detected with electrochemical luminescence (CEA assay kit, CA125 quantitative determination kit and Non-small cell lung cancer associated antigen 21-1 detection kit; Roche), whereas SCC-Ag was measured with chemiluminescent microparticle immunoassay (Architect SCC reagent kit; Abbott). According to instructions of manufacturers, the reference range was 0–5.0 ng/ml for CEA, 0.1–35.0 ng/ml for CA125, 0.1–4.0 ng/ml for CYFRA21-1, and 0–1.8 ng/ml for SCC-Ag. Lab test results and levels of serum tumor markers were categorized by low, normal, and high based on the reference range, respectively (Supplementary Table 1). PD-L1 expression was evaluated by immunohistochemistry and tumor proportion score using PD-L1 antibody (Dako 22C3) before ICIs treatment.

The study protocol was approved by the Ethics Committee of Chinese PLA General Hospital. The study was conducted in accordance with the principles of the Declaration of Helsinki and Good Clinical Practice guidelines defined by the International Conference on Harmonization. Written informed consent was collected from all patients before enrollment.

### Statistical Analysis

A post-treatment decline in serum marker level ≥20% from baseline was considered as meaningful improvement. Two groups were subsequently divided based on whether meaningful improvements of at least two serum biomarkers or not. Optimization-based methods were utilized to balance the baseline covariates between different groups (29). A weight under the following criteria was assigned to each patient: (1) Absolute value of standardized mean difference no more than 0.15; (2) Variance ratio between 0.67 (1/1.5) and 1.5. The effective sample sizes in the weighted sample were calculated by Kish's approximate formula. Group difference in ORR was calculated by Chi-square test. Median PFS and OS were estimated by Kaplan-Meier method and their 95% confidence intervals (CIs) were constructed by Brookmeyer and Crowley method, group difference was assessed by Log-rank test. Hazard ratio (HR) with its 95% CI were calculated using Cox proportional hazards models. All statistical tests were bilateral with significance level 0.05. All analyses were performed in R, with the R packages *WeightIt* version 0.5.1 *(https://cran.r-project.org/web/packages/WeightIt/index.html)* for optimization-based methods and *survey* version 3.36 *(https://cran.r-project.org/web/packages/survey/index.html)* in the weighted sample.

## Results

### Baseline Patient Characteristics

The main clinical characteristics of all the participants at baseline were presented in Table 1. Among 308 included patients, 56.2% were adenocarcinoma (ADC), 36.7% were squamous cell carcinoma (SCC) and the rest 7.1% belong to other subtypes. According to the eighth edition TNM staging of International Lung Cancer Research Association (30), 17.2% were stage IIIB, 4.2% were stage IIIC, and 78.6% were stage IV. 52.6% of patients used the drug of Pembrolizumab, 40.6% used Nivolumab, and the remaining patients used Atelizumab or Duvalumab. The median level of serum markers at baseline was 6.2 ng/ml for CEA (range 0.5–5207.0), 36.0 ng/ml for CA125 (range 3.2–2002.0), 5.1 ng/ml for CYFRA21-1 (range 1.4–345.6), and 1.2 ng/ml for SCC-Ag (range 0.2–70.0). Proportion of patients with elevated levels of CEA, CA125, CYFRA21-1, and SCC-Ag were 54.9, 51.6, 60.4, and 29.5%, respectively.

**Table 1:** Characteristics of patients at baseline.

**Characteristics**	**No. of patients (*n* = 308)**	**Percentage (%)**
Age, median (range)	61 (33–91)	
Gender		
Male	236	76.6
Female	72	23.4
Histological type		
Adenocarcinoma	173	56.2
Squamous	113	36.7
Others	22	7.1
Clinical stage		
IIIB	53	17.2
IIIC	13	4.2
IV	242	78.6
Smoking history		
Never smoker	116	37.7
Smoker or ex-smoker	192	62.3
Treatment type		
Monotherapy	149	48.4
Combination therapy	159	51.6
ECOG PS		
0–1	276	89.6
≥2	32	14.4
Prior lines of therapy		
1 line	100	32.5
2 lines	109	35.4
≥3 lines	99	32.1
Radiation history		
Yes	201	65.3
No	107	34.7
Metastasis sites		
Liver	33	10.7
Lung	102	33.1
Brain	53	17.2
Drug		
Pembrolizumab	162	52.6
Nivolumab	125	40.6
Atelizumab	8	2.6
Duvalumab	13	4.2
CEA (ng/ml)		
Median (range)	6.2 (0.5–5207.0)	
Normal (≤5.0)	139	45.1
High (>5.0)	169	54.9
CA125 (ng/ml)		
Median (range)	36.0 (3.2–2002.0)	
Normal (≤35.0)	149	48.4
High (>35.0)	159	51.6
CYFRA21-1 (ng/ml)		
Median (range)	5.1 (1.4–345.6)	
Normal (≤4.0)	122	39.6
High (>4.0)	186	60.4
SCC-Ag (ng/ml)		
Median (range)	1.2 (0.2–70.0)	
Normal (≤1.8)	217	70.5
High (>1.8)	91	29.5

*ECOG PS, Eastern Cooperative Oncology Group Performance Status; CEA, Carcinoembryonic antigen; CA125, Cancer antigen125; CYFRA21-1, Cytokeratin 19 fragment; SCC-Ag, Squamous-cell carcinoma-related antigen*.

### Association Between Dynamics of Tumor Markers and Clinical Outcomes

#### The Total Population

The total population was divided into two groups by meaningful improvements in <2 out of 4 biomarkers (CEA, CA125, CYFRA21-1, and SCC-Ag) (“<2/4 biomarkers improvement group”) and at least 2 out of 4 biomarkers (“≥2/4 biomarkers improvement group”). Standardized mean difference values of treatment type (combination therapy) and prior lines of therapy (one line, two lines) before balancing was 0.25, 0.24, and 0.18, respectively, followed by optimization-based weighting procedure to balance all baseline covariates between the two groups (Supplementary Table 2).

**Figure 1:**
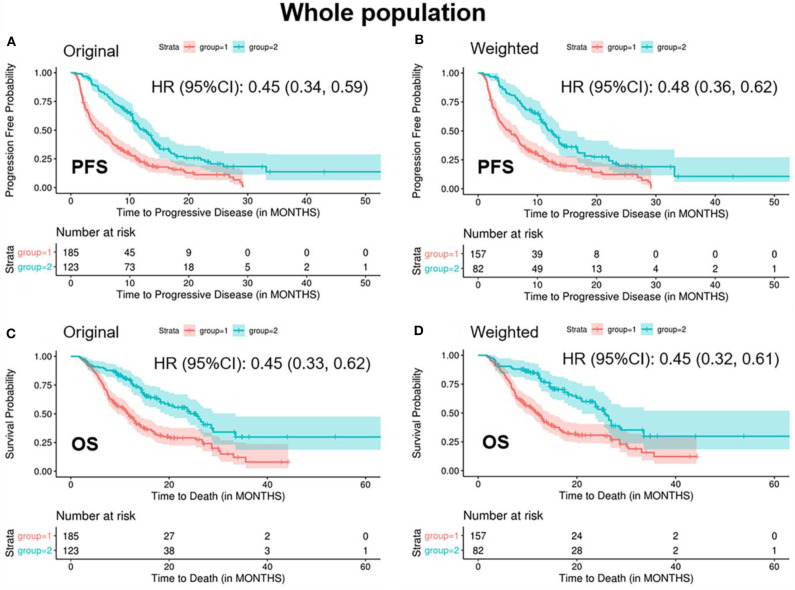
Kaplan-Meier curves of PFS/OS in the original and weighted sample of whole population. group 1: meaningful improvements in <2 out of 4 biomarkers (CEA, CA125, CYFRA21-1, and SCC-Ag); group 2: meaningful improvements in ≥2 out of 4 biomarkers (CEA, CA125, CYFRA21-1, and SCC-Ag). Kaplan-Meier curves of **(A,C)** were based on the original sample; Kaplan-Meier curves of **(B,D)** were based on the weighted sample.

In the weighted samples, the ORR in the “<2/4 biomarker improvement group” was significantly lower than the “≥2/4 biomarkers improvement group” (0.08 vs. 0.35, *p* < 0.001) (Table 2). The patients in the “<2/4 biomarker improvement group” also had significantly shorten PFS (median: 5.4 vs. 12.5 months, *p* < 0.001) and OS (median: 11.7 vs. 25.6 months, *p* < 0.001) compared with the “≥2/4 biomarkers improvement group.” The Kaplan-Meier curves of PFS and OS in both original and weighted sample were presented in Figure 1.

**Table 2:** ORR in the whole weighted sample by groups.

	**Group**	**Actual size**	**Effective size**	**Estimated ORR**	**95% CI**	***P*-value**
ORR	1	185	157	0.07	0.04–0.12	<0.001
	2	123	82	0.36	0.25–0.45	

*Group 1, meaningful improvements in <2 out of 4 biomarkers (CEA, CA125, CYFRA21-1, and SCC-Ag); Group 2, meaningful improvements in ≥2 out of 4 biomarkers (CEA, CA125, CYFRA21-1, and SCC-Ag). ORR, objective response ratio*.

#### Subgroup Analysis of ADC

**Figure 2:**
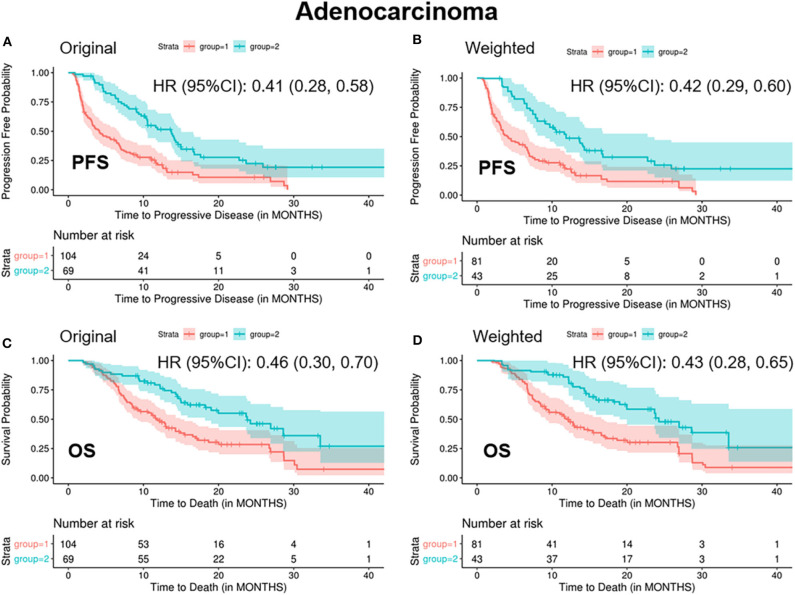
Kaplan-Meier curves of PFS/OS in the original and weighted sample of adenocarcinoma. group 1: meaningful improvements in <2 out of 4 biomarkers (CEA, CA125, CYFRA21-1, and SCC-Ag); group 2: meaningful improvements in ≥2 out of 4 biomarkers (CEA, CA125, CYFRA21-1, and SCC-Ag). Kaplan-Meier curves of **(A,C)** were based on the original sample; Kaplan-Meier curves of **(B,D)** were based on the weighted sample.

In patients with ADC, standardized mean difference of treatment type (combination therapy), prior lines of therapy (one line), and platelets (high level) was 0.25, 0.21, and 0.16, respectively, between the two groups before balancing (Supplementary Table 3). After balancing by the optimization-based method, patients in the “<2/4 biomarkers improvement group” were less likely to respond to treatment (ORR: 0.06 vs. 0.36, *p* < 0.001), more likely to progress (median PFS: 4.1 vs. 11.9 months, *p* < 0.001) and decease (median OS: 11.9 vs. 24.2 months, *p* < 0.001) (Table 3 and Figure 2).

**Table 3:** ORR in sub-populations of ADC and SCC by groups.

**Histological type**	**Group**	**Actual size**	**Effective size**	**Estimated ORR**	**95% CI**	***P*-value**
ADC	1	104	81	0.06	0.01–0.12	<0.001
	2	69	43	0.36	0.22–0.50	
SCC	1	68	47	0.08	0.01–0.16	0.014
	2	45	14	0.42	0.16–0.68	

*Group 1, meaningful improvements in <2 out of 4 biomarkers (CEA, CA125, CYFRA21-1, and SCC-Ag); Group 2, meaningful improvements in ≥2 out of 4 biomarkers (CEA, CA125, CYFRA21-1, and SCC-Ag). ADC, adenocarcinoma; SCC, squamous cell carcinoma; ORR, objective response ratio*.

#### Subgroup Analysis of SCC

**Figure 3:**
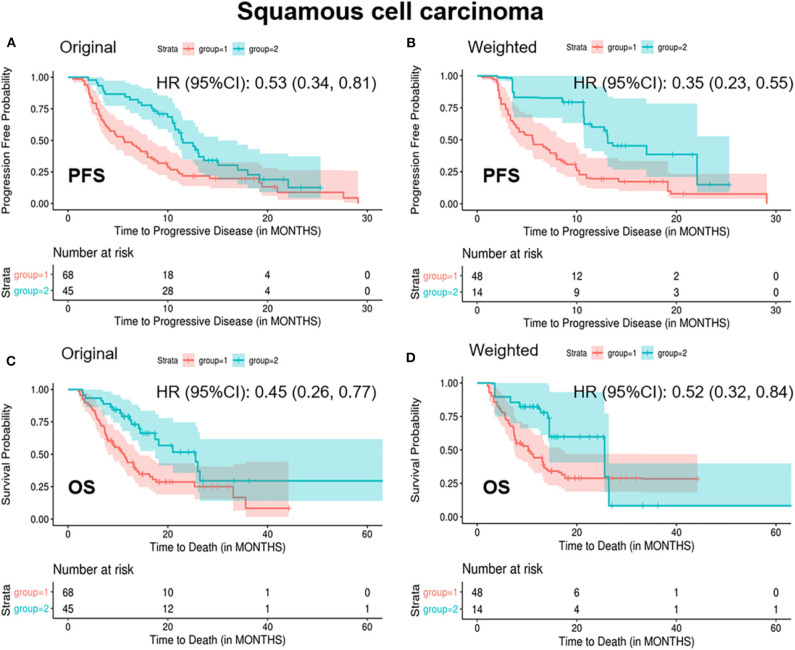
Kaplan-Meier curves of PFS/OS in the original and weighted sample of squamous cell carcinoma. group 1: meaningful improvements in <2 out of 4 biomarkers (CEA, CA125, CYFRA21-1, and SCC-Ag); group 2: meaningful improvements in ≥2 out of 4 biomarkers (CEA, CA125, CYFRA21-1, and SCC-Ag). Kaplan-Meier curves of **(A,C)** were based on the original sample; Kaplan-Meier curves of **(B,D)** were based on the weighted sample.

In patients with SCC, standardized mean difference of the baseline covariates stage (IV), treatment type (combination therapy), prior lines of therapy (one line, two lines), and radiation history (yes) before balancing was 0.16, 0.26, 0.29, 0.34, and 0.19, respectively (Supplementary Table 4). After balancing by the optimization-based method, patients in the “<2/4 biomarkers improvement group” were less likely to respond to treatment (ORR: 0.08 vs. 0.42, *p* = 0.014), more likely to progress (median PFS: 5.6 vs. 13.1 months, *p* = 0.001) and decease (median OS: 10.2 vs. 25.6 months, *p* = 0.06) (Table 3 and Figure 3).

### Association Between Dynamics of Tumor Markers and PD-L1 Expression

PD-L1 expression was measured before ICIs treatment in 70 patients, of which 44 (62.8%) were diagnosed with ADC and 26 (37.2%) were SCC. Overall, there were 12 (17.1%) patients with PD-L1 expression negative, 25 (35.7%) patients with PD-L1 expression 1–50%, and 33 (47.1%) patients with PD-L1 expression >50%. However surprisingly, our analysis showed no correlations of PD-L1 expression with dynamics of tumor markers, either in the whole group or any subgroups.

## Discussion

Recently, immune checkpoint inhibitors such as PD-1/PD-L1 inhibitors, have been widely used for advanced-stage cancer treatment. Despite of enormous success in treatment of NSCLC (31), not all patients could get long-term benefit from the treatment of ICIs (11). PD-L1 expression and TMB have been widely used as predictive markers, but their roles are still controversial (32). Reliable markers remain to be detected to identify patients who would get benefit from ICIs treatment.

In this study, we evaluated the baseline levels as well as post-treatment changes of routinely measured serum tumor markers in clinical practice to explore their associations with response to ICB therapy in patients with late-stage NSCLC. We demonstrated that dynamic changes of CEA, CA125, CYFRA21-1, and SCC-Ag were associated with the efficacy and prognosis of late-stage NSCLC patients treated with PD-1/PD-L1 inhibitors. Similar results were also observed in the subsequent subgroup analysis on ADC and SCC. Therefore, monitoring the changes in levels of serum tumor markers could be a promising prognostic factor for advanced NSCLC patients with ICIs treatment.

The approach of monitoring dynamic changes of serum tumor markers is more convenient and affordable compared to the most adopted PD-L1 expression or TMB. In contrast to other non-invasive biomarkers like lactate dehydrogenase (LDH) and neutrophil-to-lymphocyte ratio (NLR) (33–36), dynamics of serum tumor markers were also found to be more remarkably associated with response and survival according to our results, and this could also be supported by two recent studies (37, 38). Overall, as far as we know, this is the first and largest cohort study evaluating the relationship of routinely measured serum tumors markers with the efficacy and prognosis of patients receiving ICB therapy.

Optimization-based methods were used in our study. It considered the balance of baseline covariates between two groups compared to inverse propensity score weighting methods, in which only the balance of propensity score was considered in the algorithm. After balancing baseline covariates, possible confounding effects from clinical characteristics could be avoided and the collinearity in baseline covariates could also be controlled. Of noted, this is the first application of this novel statistical method in the clinical observational study.

Although we balanced all measurable baseline variates to avoid bias, there were still some limitations in our study. Firstly, the results may be influenced by the method used for choosing the cut-off point. Twenty percent was selected as a threshold to identify meaningful change in biomarkers according to previous reports, and meaningful improvement in at least two biomarkers was considered as a prognostic factor which was not data-driven. Secondly, only patients receiving more than 6 weeks of ICB treatment were enrolled in this study with baseline and post-treatment serum markers been measured, which may increase selective bias. Thirdly, dynamic change of baseline and after 6 weeks' tumor levels were used for our analysis, whether a shorter interval time is better need further investigation. Fourthly, this observational study was based on the single institution which may cause selection bias. Fifthly, we used the methods of electrochemical luminescence and chemiluminescent microparticle immunoassay for testing tumor markers, some new methods with high sensitivity and specificity may be more helpful for early detection of tumor markers (39, 40). Last but not least, though weighting method were used to balance all measurable baseline covariates, some unrecorded baseline covariates such as TMB could be potential confounders.

## Conclusions

In summary, we proposed a new strategy of monitoring dynamics of serum tumor markers and highlight their importance as a potential prognostic biomarker of advanced NSCLC treated with ICIs. Decrease of associated biomarkers serum levels were associated with favorable clinical outcomes. Further investigations will be required to evaluate the roles of these serum markers with different cut-off values as well as earlier dynamic changes from baseline in larger multi-center patient populations.

## Data Availability Statement

All datasets generated for this study are included in the article/Supplementary Material.

## Ethics Statement

The studies involving human participants were reviewed and approved by People's Liberation Army General Hospital. The patients/participants provided their written informed consent to participate in this study.

## Author Contributions

ZZ, FY, RC, YL, JM, XY, LW, FZ, HT, DG, ZH, SZ, XL, XZ, XG, YH, and JW contributed to the study design. YH and JW were responsible for interpretation of the results. DG and ZH contributed to statistical analysis. ZZ, FY, and RC were prepared for the manuscript. All authors contributed to data analysis.

## Conflict of Interest

The authors declare that the research was conducted in the absence of any commercial or financial relationships that could be construed as a potential conflict of interest. The reviewer YM declared a shared affiliation, with no collaboration, with one of the authors, RC, to the handling editor at the time of review.

## Abbreviations

NSCLC: non-small cell lung cancer
ADC: adenocarcinoma
SCC: squamous cell carcinoma
CA125: cancer antigen 125
CEA: carcinoembryonic antigen
CYFRA21-1: cytokeratin 19 fragment
SCC-Ag: squamous-cell carcinoma-related antigen
ICB: immune checkpoint blockade
ICIs: immune checkpoint inhibitors
CR: complete response
PR: partial response
SD: stable disease
PD: progressive disease
ORR: objective response rate
PFS: progression-free survival
OS: overall survival
PD-1/PD-L1: programmed cell death-1/programmed cell death ligand-1
RECIST: Response Evaluation Criteria in Solid Tumors
TMB: tumor mutation burden.
