# Supplementary material for: Dynamics of Serum Tumor Markers Can Serve as a Prognostic Biomarker for Chinese Advanced Non-small Cell Lung Cancer Patients Treated With Immune Checkpoint Inhibitors
Source: Front Immunol. 2020 Jun 10;11:1173. doi: 10.3389/fimmu.2020.01173 (PMC7298878; doi:10.3389/fimmu.2020.01173)
Supplement: Supplementary file 1 [file Data_Sheet_1.docx]

**Supplements**

**Supplementary Table 1** Normal range of lab variables.

| **Lab variables** | **LLN** | **ULN** | **Unit** |
| --- | --- | --- | --- |
| HGB Male | 137 | 179 | g/L |
| Female | 116 | 155 | g/L |
| WBC | 3.5 | 10.0 | 10^9/L |
| NEUT | 0.5 | 0.7 | % |
| LY | 0.2 | 0.4 | % |
| MONO | 0.03 | 0.08 | % |
| PLT | 100 | 300 | 10^9/L |
| ALB | 35 | 50 | g/L |
| LDH | 40 | 250 | U/L |
| CEA | 0 | 0.5 | ug/L |
| CA125 | 0.1 | 35.0 | u/mL |
| CYFRA21-11 | 0.1 | 4.0 | ng/mL |
| SCC-Ag | 0 | 1.8 | ng/mL |

LLN: lower limits of normal; ULN: upper limits of normal; HGB: hemoglobin; WBC: white blood cell; NEUT: neutrophil; LY: Lymphocyte; MONO: monocyte; PLT: Platelet; ALB: Albumin; LDH: Lactic dehydrogenase; CEA: Carcinoembryonic antigen; CA125: Cancer antigen125; CYFRA21-1: Cytokeratin 19 fragment; SCC-Ag: Squamous-cell carcinoma-related antigen.

**Supplementary Table 2** Baseline characteristics balanced between the two groups.

| Characteristics | Type | Before balancing | | After balancing | |
| --- | --- | --- | --- | --- | --- |
|  |  | **Difference** | **Variance Ratio** | **Difference** | **Variance Ratio** |
| Age (year) | Contin | 0.11 | 1.04 | 0.00 | 0.98 |
| Gender |  |  |  |  |  |
| Male | Binary | 0.04 |  | 0.00 |  |
| Female | Binary | -0.04 |  | 0.00 |  |
| Histological type |  |  |  |  |  |
| Adenocarcinoma | Binary | 0.00 |  | 0.00 |  |
| Squamous | Binary | 0.00 |  | 0.00 |  |
| Others | Binary | 0.00 |  | 0.00 |  |
| Clinical stage |  |  |  |  |  |
| IIIB/C | Binary | -0.05 |  | 0.00 |  |
| IV | Binary | 0.05 |  | 0.00 |  |
| Smoking history |  |  |  |  |  |
| Yes | Binary | 0.02 |  | 0.00 |  |
| No | Binary | -0.02 |  | 0.00 |  |
| ECOG PS |  |  |  |  |  |
| 0-1 | Binary | -0.02 |  | 0.00 |  |
| ≥ 2 | Binary | 0.02 |  | 0.00 |  |
| Treatment type |  |  |  |  |  |
| Monotherapy | Binary | -0.25 |  | 0.00 |  |
| Combination therapy | Binary | 0.25 |  | 0.00 |  |
| Prior lines of therapy |  |  |  |  |  |
| 1 | Binary | 0.24 |  | 0.00 |  |
| 2 | Binary | -0.18 |  | 0.00 |  |
| ≥ 3 | Binary | -0.06 |  | 0.00 |  |
| Radiation history |  |  |  |  |  |
| Yes | Binary | -0.14 |  | 0.00 |  |
| No | Binary | 0.14 |  | 0.00 |  |
| Metastasis sites | | | | | |
| Liver | Binary | 0.03 |  | 0.00 |  |
| Lung | Binary | -0.01 |  | 0.00 |  |
| Brain | Binary | -0.04 |  | 0.00 |  |
| WBC |  |  |  |  |  |
| Low | Binary | -0.05 |  | 0.00 |  |
| Normal | Binary | 0.03 |  | 0.00 |  |
| High | Binary | 0.02 |  | 0.00 |  |
| HGB |  |  |  |  |  |
|  | Contin | 0.05 | 1.14 | 0.00 | 1.05 |
| NEUT |  |  |  |  |  |
| Low | Binary | -0.04 |  | 0.00 |  |
| Normal | Binary | -0.04 |  | 0.00 |  |
| High | Binary | 0.08 |  | 0.00 |  |
| LY |  |  |  |  |  |
| Low | Binary | 0.03 |  | 0.00 |  |
| Normal | Binary | -0.01 |  | 0.00 |  |
| High | Binary | -0.02 |  | 0.00 |  |
| MONO |  |  |  |  |  |
| Low | Binary | 0.03 |  | 0.00 |  |
| Normal | Binary | 0.04 |  | 0.00 |  |
| High | Binary | -0.07 |  | 0.00 |  |
| PLT |  |  |  |  |  |
| Low | Binary | -0.02 |  | 0.00 |  |
| Normal | Binary | -0.09 |  | 0.00 |  |
| High | Binary | 0.11 |  | 0.00 |  |
| ALB |  |  |  |  |  |
| Normal | Binary | 0.02 |  | 0.00 |  |
| High | Binary | -0.02 |  | 0.00 |  |
| LDH |  |  |  |  |  |
| Normal | Binary | -0.01 |  | 0.00 |  |
| High | Binary | 0.01 |  | 0.00 |  |
| CEA |  |  |  |  |  |
| Normal | Binary | -0.01 |  | 0.00 |  |
| High | Binary | 0.01 |  | 0.00 |  |
| CA125 |  |  |  |  |  |
| Normal | Binary | -0.07 |  | 0.00 |  |
| High | Binary | 0.07 |  | 0.00 |  |
| CYFRA21-1 |  |  |  |  |  |
| Normal | Binary | -0.08 |  | 0.00 |  |
| High | Binary | 0.08 |  | 0.00 |  |
| SCC-Ag |  |  |  |  |  |
| Normal | Binary | -0.13 |  | 0.00 |  |
| High | Binary | 0.13 |  | 0.00 |  |

Contin: continuous variable; Binary: binary variable; ECOG PS: Eastern Cooperative Oncology Group Performance Status; WBC: white blood cell; HGB: hemoglobin; NEUT: neutrophil; MONO: monocyte; PLT: Platelet; ALB: Albumin; CEA: Carcinoembryonic antigen; CA125: Cancer antigen125; CYFRA21-1: Cytokeratin 19 fragment; SCC-Ag: Squamous-cell carcinoma-related antigen.

**Supplementary Table 3** Baseline characteristics of patients with adenocarcinoma.

| Characteristics | Type | Before balancing | | After balancing | |
| --- | --- | --- | --- | --- | --- |
|  |  | **Difference** | **Variance Ratio** | **Difference** | **Variance Ratio** |
| Age (y) | Contin | -0.02 | 1.044 | 0.00 | 0.958 |
| Gender |  |  |  |  |  |
| Male | Binary | 0.05 |  | 0.00 |  |
| Female | Binary | -0.05 |  | 0.00 |  |
| Clinical stage |  |  |  |  |  |
| IIIB/C | Binary | 0.04 |  | 0.00 |  |
| IV | Binary | -0.04 |  | 0.00 |  |
| Smoking history |  |  |  |  |  |
| Yes | Binary | 0.06 |  | 0.00 |  |
| No | Binary | -0.06 |  | 0.00 |  |
| ECOG PS |  |  |  |  |  |
| 0-1 | Binary | -0.02 |  | 0.00 |  |
| ≥ 2 | Binary | 0.02 |  | 0.00 |  |
| Treatment |  |  |  |  |  |
| Monotherapy | Binary | -0.25 |  | 0.00 |  |
| Combination therapy | Binary | 0.25 |  | 0.00 |  |
| Prior lines of therapy |  |  |  |  |  |
| 1 | Binary | 0.21 |  | 0.00 |  |
| 2 | Binary | -0.08 |  | 0.00 |  |
| ≥ 3 | Binary | -0.13 |  | 0.00 |  |
| Radiation history |  |  |  |  |  |
| Yes | Binary | -0.08 |  | 0.00 |  |
| No | Binary | 0.08 |  | 0.00 |  |
| Metastasis sites |  |  |  |  |  |
| Liver | Binary | -0.01 |  | 0.00 |  |
| Lung | Binary | 0.02 |  | 0.00 |  |
| Brain | Binary | -0.05 |  | 0.00 |  |
| WBC |  |  |  |  |  |
| Low | Binary | -0.05 |  | 0.00 |  |
| Normal | Binary | 0.06 |  | 0.00 |  |
| High | Binary | -0.01 |  | 0.00 |  |
| HGB |  |  |  |  |  |
|  | Contin | 0.01 | 1.14 | 0.00 | 1.01 |
| NEUT |  |  |  |  |  |
| Low | Binary | -0.05 |  | 0.00 |  |
| Normal | Binary | -0.07 |  | 0.00 |  |
| High | Binary | 0.13 |  | 0.00 |  |
| LY |  |  |  |  |  |
| Low | Binary | 0.05 |  | 0.00 |  |
| Normal | Binary | -0.02 |  | 0.00 |  |
| High | Binary | -0.03 |  | 0.00 |  |
| MONO |  |  |  |  |  |
| Low | Binary | 0.03 |  | 0.00 |  |
| Normal | Binary | 0.07 |  | 0.00 |  |
| High | Binary | -0.10 |  | 0.00 |  |
| PLT |  |  |  |  |  |
| Low | Binary | -0.01 |  | 0.00 |  |
| Normal | Binary | -0.15 |  | 0.00 |  |
| High | Binary | 0.16 |  | 0.00 |  |
| ALB |  |  |  |  |  |
| Normal | Binary | -0.05 |  | 0.00 |  |
| High | Binary | 0.05 |  | 0.00 |  |
| LDH |  |  |  |  |  |
| Normal | Binary | -0.04 |  | 0.00 |  |
| High | Binary | 0.04 |  | 0.00 |  |
| CEA |  |  |  |  |  |
| Normal | Binary | 0.12 |  | 0.00 |  |
| High | Binary | -0.12 |  | 0.00 |  |
| CA125 |  |  |  |  |  |
| Normal | Binary | -0.04 |  | 0.00 |  |
| High | Binary | 0.04 |  | 0.00 |  |
| CYFRA21-1 |  |  |  |  |  |
| Normal | Binary | -0.05 |  | 0.00 |  |
| High | Binary | 0.05 |  | 0.00 |  |
| SCC-Ag |  |  |  |  |  |
| Normal | Binary | -0.12 |  | 0.00 |  |
| High | Binary | 0.12 |  | 0.00 |  |

Contin: continuous variable; Binary: binary variable; ECOG PS: Eastern Cooperative Oncology Group Performance Status; WBC: white blood cell; HGB: hemoglobin; NEUT: neutrophil; MONO: monocyte; PLT: Platelet; ALB: Albumin; CEA: Carcinoembryonic antigen; CA125: Cancer antigen125; CYFRA21-1: Cytokeratin 19 fragment; SCC-Ag: Squamous-cell carcinoma-related antigen.

**Supplementary Table 4** Baseline characteristics of patients with squamous cell carcinoma.

| Characteristics | Type | Before balancing | | After balancing | |
| --- | --- | --- | --- | --- | --- |
|  |  | **Difference** | **Variance Ratio** | **Difference** | **Variance Ratio** |
| Age (y) | Contin | 0.12 | 0.86 | 0.06 | 1.01 |
| Gender |  |  |  |  |  |
| Male | Binary | -0.001 |  | 0.02 |  |
| Female | Binary | 0.001 |  | -0.02 |  |
| Clinical stage |  |  |  |  |  |
| IIIB/C | Binary | -0.16 |  | -0.03 |  |
| IV | Binary | 0.16 |  | 0.03 |  |
| Smoking history |  |  |  |  |  |
| Yes | Binary | -0.05 |  | 0.01 |  |
| No | Binary | 0.05 |  | -0.01 |  |
| ECOG PS |  |  |  |  |  |
| 0-1 | Binary | -0.02 |  | 0.00 |  |
| ≥ 2 | Binary | 0.02 |  | 0.00 |  |
| Treatment |  |  |  |  |  |
| Monotherapy | Binary | -0.26 |  | -0.05 |  |
| Combination therapy | Binary | 0.26 |  | 0.05 |  |
| Prior lines of therapy |  |  |  |  |  |
| 1 | Binary | 0.30 |  | 0.06 |  |
| 2 | Binary | -0.35 |  | -0.07 |  |
| ≥ 3 | Binary | 0.05 |  | 0.01 |  |
| Radiation history |  |  |  |  |  |
| Yes | Binary | -0.19 |  | -0.06 |  |
| No | Binary | 0.19 |  | 0.06 |  |
| Metastasis sites |  |  |  |  |  |
| Liver | Binary | 0.02 |  | -0.02 |  |
| Lung | Binary | -0.03 |  | -0.02 |  |
| Brain | Binary | -0.01 |  | 0.04 |  |
| WBC |  |  |  |  |  |
| Low | Binary | -0.07 |  | -0.01 |  |
| Normal | Binary | -0.02 |  | 0.00 |  |
| High | Binary | 0.09 |  | 0.01 |  |
| HGB |  |  |  |  |  |
|  | Contin | 0.05 | 1.21 | 0.02 | 1.02 |
| NEUT |  |  |  |  |  |
| Low | Binary | -0.02 |  | -0.01 |  |
| Normal | Binary | -0.01 |  | -0.01 |  |
| High | Binary | 0.03 |  | 0.02 |  |
| LY |  |  |  |  |  |
| Low | Binary | 0.01 |  | 0.01 |  |
| Normal | Binary | 0.01 |  | 0.01 |  |
| High | Binary | -0.02 |  | -0.02 |  |
| MONO |  |  |  |  |  |
| Low | Binary | -0.00 |  | 0.00 |  |
| Normal | Binary | 0.05 |  | 0.05 |  |
| High | Binary | -0.05 |  | -0.05 |  |
| PLT |  |  |  |  |  |
| Low | Binary | -0.01 |  | 0.00 |  |
| Normal | Binary | 0.03 |  | 0.03 |  |
| High | Binary | -0.02 |  | -0.03 |  |
| ALB |  |  |  |  |  |
| Normal | Binary | 0.03 |  | 0.01 |  |
| High | Binary | -0.03 |  | -0.01 |  |
| LDH |  |  |  |  |  |
| Normal | Binary | 0.01 |  | 0.01 |  |
| High | Binary | -0.01 |  | -0.01 |  |
| CEA |  |  |  |  |  |
| Normal | Binary | -0.11 |  | -0.03 |  |
| High | Binary | 0.11 |  | 0.03 |  |
| CA125 |  |  |  |  |  |
| Normal | Binary | -0.10 |  | -0.02 |  |
| High | Binary | 0.10 |  | 0.02 |  |
| CYFRA21-1 |  |  |  |  |  |
| Normal | Binary | -0.06 |  | -0.01 |  |
| High | Binary | 0.06 |  | 0.01 |  |
| SCC-Ag |  |  |  |  |  |
| Normal | Binary | -0.11 |  | -0.07 |  |
| High | Binary | 0.11 |  | 0.07 |  |

Contin: continuous variable; Binary: binary variable; ECOG PS: Eastern Cooperative Oncology Group Performance Status; WBC: white blood cell; HGB: hemoglobin; NEUT: neutrophil; MONO: monocyte; PLT: Platelet; ALB: Albumin; CEA: Carcinoembryonic antigen; CA125: Cancer antigen125; CYFRA21-1: Cytokeratin 19 fragment; SCC-Ag: Squamous-cell carcinoma-related antigen.
